# National Survey on the importance of sleep in the quality of academic life and mental health of college students in Portugal

**DOI:** 10.5935/1984-0063.20200090

**Published:** 2021

**Authors:** Marta Reis, Lúcia Ramiro, Teresa Paiva, Margarida Gaspar-de-Matos

**Affiliations:** 1 Faculdade de Motricidade Humana/Universidade de Lisboa, Lisboa, Portugal.; 2 ISAMB/Faculdade de Medicina, Universidade de Lisboa, Lisboa, Portugal.; 3 CENC - Sleep Medicine Center, Lisboa, Portugal.

**Keywords:** Sleep, Quality of academic life, Mental Health, College students, Portugal

## Abstract

The academic years are a period of vulnerability when considering sleep problems and mental health. Growing evidence suggests poor sleep patterns are related to impaired academic life and lower psychological well-being. The aim of this study was to explore the importance of sleep habits and report the associations of sleep problems with quality of academic life and different dimensions of mental health (e.g. worries, anxiety, self-regulation and resilience) in a large sample of college students. The HBSC/JUnP data base comprises a representative sample of 2991 college students (n=2203; 73.7% women), aged from 18 to 35 years old (22.43±3.83). Participants socio-demographic characteristics included sex and age. Besides, participants were inquired about sleep duration, characteristics of their sleep habits, questions about quality academic life, namely feeling bored in university, pressure from academic work and academic performance perception and mental health, namely worries, anxiety, self-regulation and resilience. Results showed most young people report an average value for sleep habits (M=4.41; SD=1.46) and that they sleep an average of 7 hours a night. More than half of the young people report either being affected by difficulty falling asleep, showing signs of sleep onset insomnia (67.7%). The conducted analyses indicated that the difficulty falling asleep (having insomnia) was associated with poor/reasonable academic performance perception, higher levels of concerns and anxiety, and lower levels of self-regulation and resilience, thus jeopardizing the mental health of college students. In turn, that characteristics of sleep was also associated with poor sleep habits. In conclusion, this study showed that poor sleep habits were associated with a worse level of academic performance perception and low levels of mental health among college students in Portugal. Universities offer enormous potential as settings to promote sleep-health programs since they can reach many young people who are future-oriented and willing to learn. There is then the need for academic researchers, teaching staff and health professionals working for college students health, to develop and test a wide array of sleep-promoting interventions (e.g., education classes, online programs, adjustment of class time), thus preventing negative secondary outcomes.

## INTRODUCTION

The academic years are a period of vulnerability when considering sleep problems and mental health^1^. Growing evidence suggests poor sleep patterns are related to impaired academic performance^2,3^ and lower psychological well-being^4,5^.

For older adults (46-60 years), the recommended sleep duration is 7h or more per night on a regular basis; for younger adults (18-45 years), an even longer duration (>9) is considered appropriate^6^. Sleep greatly influences mental function, and thus, affects performance. Insufficient sleep decreases general alertness, impairs attention, and slows cognitive processing^7^.

Sleep problems tend to worsen over time in university students, a finding that is alarming since significant sleep problems are associated with reduced mental health outcomes^1^.

With regard to the academic performance, Gomes et al. (2011)^3^ demonstrated that both sleep duration and quality are significant predictors of school grades among college students. In other study with college students, Kelly et al. (2001)^8^ found a positive correlation between sleep duration and school grades. Students with ≥8h of sleep reported a grade-point-average (GPA) of 3.24 compared to an average GPA of 2.74 for those with 7h of sleep. Similar results were obtained in technological/engineering students of a Portuguese University^9^. Psycho-physiological research indicated that sleep is crucial for the consolidation and reactivation of memory^10^. Sleep-deprived participants have greater difficulty than healthy participants (control group) in recalling learned materials. Good sleep quality was also associated with higher learning motivation and school performance^5,11,12^.

In terms of mental health, approximately 25% of university students who did not report a mental health issue at a baseline assessment ultimately reported a mental health problem 2 years later; approximately 60% of students in that study experienced continuity in the report of at least one mental health issue over time. Approximately 50% of the students who reported continuous mental health issues across assessments did not seek mental health treatment^13^. Although the studies have established that sleep problems are associated with poor mental health at all ages^14^, there is a well-known high prevalence of mental health problems in university students, including high levels of perceived stress^15^, anxiety^16^ and depressive symptoms^17^.

Therefore, in this paper, we assessed sleep habits, quality of academic life, and mental health in a national sample of college students in Portugal. The aim of this study was to explore the importance of sleep habits and report the associations of sleep problems (e.g., difficulty falling asleep [insomnia]) with quality of academic life, namely academic performance perception and different dimensions of mental health (e.g., worries, anxiety, self-regulation, and resilience) in a large sample of female and male university students from different years and fields of study.

## MATERIAL AND METHODS

### Study design, participants and procedures

The national HBSC/JUnP study is an extension of two investigations conducted by the Social Adventure team - HBSC/WHO (Health Behavior in school aged children; http://aventurasocial.com/verartigo.php?article_id=238) and HBSC/SSREU (Sexual and Reproductive Health of university students; http://aventurasocial.com/verartigo.php?article_id=98). The HBSC/JUnP followed all the rules for research outlined in 2008 by the World Medical Association Declaration of Helsinki, and was approved by the Ethics Commission of the Medicine Academic Center of Lisbon of the Faculty of Medicine, University of Lisbon.

The HBSC/JUnP data base comprises a representative sample of 2,991 college students (n=2203; 73.7% women), aged from 18 to 35 years old (22.43±3.83). The distribution according to the age groups is as follows: 18-20 years old (n=1096; 36.6%), 21-23 years old (n=1073; 35.9%), 24-35 years old (n=822; 27.5%). It provides national representative data from those attending university during the academic year of 2015/2016. Data were collected through a self-administered questionnaire. The sample included 73.7% women and 26.3% men (this percentage is consistent with the gender distribution in the Portuguese university population: DGES/MCTES, 2016). The majority of students has Portuguese nationality (95.6%), is single (92.3%) and was proportionally distributed among all the educational Portuguese regions.

Data collection was based on an online cross-sectional survey, made available on the *Limesurvey* platform, and administered to college students in Portugal. The students could access the survey with whatever electronic device that was capable of accessing their email. The survey began with a brief description of the study itself, followed by a detailed informed consent that explained the participants' rights, the perceived risks and benefits of participation, and the measures taken by the researchers to keep their personal information confidential. The students could access the survey only after they agreed to this informed consent. It took a participant around 25/30 minutes to fill out the survey.

### Measures

For the purpose of this study, the following variables were selected: participants' sociodemographic characteristics included sex and age. Sleep status was measured based on four parameters: (1) duration of sleep at weeknights (measured in hours per night), (2) duration of sleep at weekend nights (measured in hours per night), (both categorized between 4h or less and 11h or more); (3) quality of sleep - whether they consider they sleep well, whether they sleep too much, sleep too little, restless sleep, difficulty waking up in the morning, difficulty falling asleep, waking up in the middle of the night, and waking up in the morning before the alarm clock, (categorized as never/almost never, sometimes, and almost always/always); and (4) sleep habits measured by the sum of the following 7 items: sleeping too much, sleeping too little, restless sleep, difficulty waking up in the morning, difficulty falling asleep, waking up in the middle of the night, and waking up in the morning before the alarm clock. Each item was recoded between 0 (never/almost never) and 1 (sometimes and almost always/always). Regarding the average sleep habits, the results obtained can vary between 0 and 7 points, the highest value being an indicator of poor sleep habits.

College students were asked about their perception of health, i.e., how they assessed their health (categorized as excellent, very good, good, reasonable, and bad).

Quality of academic life was self-assessed by taking into account three items: (1) feeling bored in university (categorized as never, sometimes, and frequently/always), (2) being under pressure from academic work (categorized as not at all/slightly, fairly, and very much) and (3) academic performance perception (categorized as poor/reasonable, good, and very good/excellent). As for their academic performance perception, response options were "poor/reasonable", "good", and "very good/excellent" and were subsequently dichotomized into "bad" ("poor/reasonable") and "good" ("good" and "very good/excellent").

Mental health was measured by two questions about frequency and intensity of worries and by three scales: (1) anxiety-STAI-T^18,19^, (2) self-regulation-SR^20^, and (3) resilience-RES^21^. For a better understanding of the measures see Table 1.

**Table 1.** Descriptive of the variables under study.

	Excellent (N/%)	Very good (N/ %)	Good (N/%)	Reasonable (N/%)	Bad (N/%)
Health perception (N=2991)	241; 8.1	1155; 38.6	1049; 35.1	491; 16.4	55; 1.8
	**M**	**SD**	**Min.**	**Max.**	
Hours of sleep at weeknights (N=2991)	7.00	1,09	4	11	
Hours of sleep at weekend nights (N=2991)	8.55	1,34	4	11	
Sleep habits (N=2991)	4.41	1,46	0	7	
	**Never/almost never (N/ %)**	**Sometimes (N/ %)**	**Almost always/always (N/ %)**		
Sleeping well (N=2991)	189; 6.3	1075; 35.9	1727; 57.7		
Sleeping too much (N=2991)	1329; 44.4	1450; 48.5	212; 7.1		
Sleeping too little (N=2991)	629; 21.0	1804; 60.3	558; 18.7		
Sleeping with agitation (N=2991)	1555; 52.0	1131; 37.8	305; 10.2		
Difficulty waking up in the morning (N=2991)	654; 21.9	1238; 41.4	1099; 36.7		
Difficulty falling asleep (N=2991)	965; 32.3	1337; 44.7	689; 23.0		
Waking up in the middle of the night (N=2991)	1365; 45.6	1202; 40.2	424; 14.2		
Waking up in the morning before the alarm clock (N=2991)	1237; 41.4	1372; 45.9	382; 12.8		
	**Several times a day (N/%)**	**Almost every day (N/%)**	**Several times a week (N/%)**	**Several times a month (N/%)**	**Rarely or never (N/%)**
Frequency of worries (N=2991)	264; 8.8	564; 18.9	1090; 36.4	827; 27.6	246; 8.2
	**So intense that they do not allow me to think of anything else (N/%)**	**They trouble me but let my life keep going (N/%)**	**I have worries but I do not let them interfere with my life (N/%)**	**I worry about nothing at all (N/%)**	
Intensity of worries (N=2991)	362; 12.1	1492; 49.9	1046; 35.0	91; 3.0	
	**M**	**DP**	**Min.**	**Max.**	
Anxiety - trait (STAI-T) (N=2991)	44,58	9,28	23	74	
Self-regulation - total (N=2991)	143,94	16,68	78	203	
Short-term self-regulation subscale (N=2991)	59,82	9,34	31	88	
Long-term self-regulation subscale (N=2991)	84,12	12,9	43	118	
Resilience (N=2991)	55.49	8.10	21	72	
	Never (N/%)	Sometimes (N/%)	Frequently/always (N/%)		
Feeling bored in University (N=2991)	256; 8.6	2086; 69.7	649; 21.7		
	Not at all/slightly (N/ %)	Fairly (N/ %)	Very much (N/ %)		
Being under pressure from academic work (N=2991)	383; 12.8	1461; 48.8	1147; 38.3		
	Poor/reasonable (N/ %)	Good (N/ %)	Very good/excellent (N/ %)		
Academic performance perception (N=2991)	833; 27.9	1654; 55.3	504; 16.9		

### Data analysis

Data from *Limesurvey* was transferred to an electronic data file. All variables were checked for data inaccuracy by running SPSS frequencies. Subsequently, an analysis on missing values was conducted. All data were tested for normality prior to any analyses using Kolmogorov-Smirnov tests, as well as Levene's test for the homogeneity of the variance. Both descriptive and inferential statistics were applied. All statistical analyses were completed using the SPSS 24.0 (Statistical Package for Social Sciences).

Difficulty falling asleep was then compared between gender, age and all variables under study, using chi-square (χ2) and ANOVA. The level of significance was set at 0.05. Only significant results were discussed. The associations between several independent variables (hours of sleep at weeknights, hours of sleep at weekend nights, sleep habits, anxiety - trait (STAI-T), self-regulation - total, long-term self-regulation subscale, resilience) and the dependent variables (having sleeping well, perception of good academic performance and having worries) were ascertained using bivariate logistic regression analysis. Odds ratios (ORs) and 95% confidence intervals (CIs) were calculated.

## RESULTS

### Descriptive of the variables under study

Table 1 shows the characteristics of the sample for the variables under study. More than two thirds of young university students consider their health to be either very good (38.6%) or good (35.1%). Regarding sleep duration, young people sleep an average of 7 hours at weeknights (SD=1.09) and 8.55 hours (SD=1.34) at weekend nights, resulting into a mean value for sleep habits of 4.41; (SD=1.46). Yet, although more than half of the participants report sleeping well almost always/always (57.7%), more than half of them report that - "sometimes" and "almost always/always" they were sleeping too little (79%), having difficulty waking up in the morning (78.1%), had difficulty falling asleep (67.7%) -, wake up in the morning before the alarm clock (58.7%), sleep too much (55.6%)", wake up in the middle of the night (54.4%), and have a restless sleep (48%).

In terms of quality of academic life the vast majority of young people admit feeling bored in the university (91.4% "sometimes" and "often/always"), being fairly or very much under pressure from academic work (87.1%) and more than a quarter of young people reported having a poor or reasonable academic performance perception (27.9%).

As for mental health issues, it was observed that only 8.2% of the participants in the study reported rarely or never feeling worried and only 3% mentioned worrying about nothing at all. The results obtained from the anxiety, self-regulation and resilience scales showed that university students in Portugal present average values ​​of anxiety (M=44.58; SD=9.28), but also mean values ​​of the self-regulation total (M=143.94; SD=16.68), in the short-term (M=59.82; SD=9.34) and in the long-term (M=84.12; SD=12.90), as well as mean values of resilience (M=55.49; SD=8.10).

### Differences between difficulty falling asleep and quality of academic life and mental health of the university students in Portugal

College students who most often report feeling "frequently/always" bored in the university (27.9%), being very much under pressure from academic work (45.4%) and having a poor/reasonable self-reported academic performance perception (37.6%) are more likely to show frequent difficulties falling asleep ("almost always/always"). In turn, those who mention never being bored in the university (10.1%), those who feel they are only fairly under pressure from academic work (51.7%) and those who think they have a very good/excellent academic performance perception (21%) are the ones who most often say they never/almost never experience difficulty falling asleep (χ2 (3) = 44.222; *p*<.001; χ2 (3) = 24.906; *p*<.001; χ2 (3) = 60.038; *p*<.001).

There are also young people who mention having worries several times a day (18%), almost every day (21.6%) and several times a month (22.9%), as well as some who say they are worried to the extent that they cannot think of anything else (18.7%) or to the extent that it troubles them but they keep going (54.1%). These are also the ones who most often report having difficulty falling asleep almost always/always. By contrast, young people who admit to rarely/never having worries (13.9%) and the ones who may even have worries but do not let them interfere with their life (40.4%) are more likely to say that they never/almost never feel difficulties falling asleep (χ2 (5) = 164.370; *p*<.001; χ2 (4) = 74.664; *p*<.001).

And finally, difficulty falling asleep had a statistically significant effect on anxiety -trait (STAI-T) (F (2, 2988) = 111.198, *p*=.000), self-regulation - total (F (2, 2988) = 75.562, *p*=.000), the short-term self-regulation subscale (F (2, 2988) = 79.127, *p*=.000), the long-term self-regulation subscale (F (2, 2988) = 37.019, *p*=.000), resilience (F (2, 2988) = 26.981, *p*=.000), hours of sleep at weeknights (F (2, 2988) = 45.616, *p*=.000), hours of sleep at weekend nights (F (2, 2988) = 4.376, *p*=.013), and sleep habits (F (2, 2988) = 855.823, *p*=.000).

The Tukey post-hoc comparisons for long-term self-regulation and sleep habits and by Games-Howell method for other variables indicated that young people with difficulty falling asleep almost always/always show relatively more signs of anxiety (M=48.81, SD=9.94), a lower self-regulation - total (M=136.77, SD=20.00), a lower capacity of self-regulation in the short-term (M=56.20, SD=9.97), a lower capacity of self-regulation in the long-term (M=80.58, SD=13.42), less resilience (M=53.68, SD=8.55), less hours of sleep at weeknights (M=6.66, SD=1.22), and worst sleep habits (M=5.34 SD=1.07), when compared with the other two groups, namely the group that reported having occasional ("sometimes") difficulties falling asleep and the group that reported never or almost never having difficulties falling asleep (Table 2).

**Table 2.** Differences between difficulty falling asleep and quality of academic life and mental health of the university students in Portugal (N=2991).

	Difficulty falling asleep								
	Never/almost never (N=965)		Sometimes (N=1337)		Almost always/always(N=689)		χ²	p	
	N	%	N	%	N	%			
							7.716	≤.050	
Gender								(p=.021)	
Male	283	**29.3**	323	24.2	182	26.4			
Female	682	70.7	1014	**75.8**	507	73.6			
							3.408	n.s.	
Age group								(p=.492)	
18-20 years old	336	34.8	512	38.3	248	36.0			
21-23 years old	360	37.3	461	34.5	252	36.6			
24-35 years old	269	27.9	364	27.2	189	27.4			
							2.466	n.s.	
Health perception								(p=.963)	
Excellent	84	8.7	106	7.6	51	7.4			
Very good	377	39.1	509	38.1	269	39.0			
Good	330	34.2	483	36.1	236	34.3			
Reasonable	157	16.3	214	16.0	120	17.4			
Bad	17	1.8	25	1.9	13	1.9			
							164.370	≤.001	
Frequency of worries								(p=.000)	
Several times a day	50	5.2	90	6.7	124	**18.0**			
Almost every day	172	17.8	243	18.2	149	**21.6**			
Several times a week	331	34.3	524	**39.2**	235	34.1			
Several times a month	278	28.8	391	29.2	158	**22.9**			
Rarely or never	134	**13.9**	89	6.7	23	3.3			
							76.664	≤.001	
Intensity of worries								(p=.000)	
So intense that they do not allow me to think of anything else	84	8.7	149	11.1	129	**18.7**			
They trouble me but I keep going	462	47.9	657	49.1	373	**54.1**			
								
I have worries but I do not let them interfere with my life	390	**40.4**	495	37.0	161	23.4			
I worry about nothing at all	29	3.0	36	2.7	26	3.8			
							44.222	≤.001	
Feeling bored in University								(p=.000)	
Never	97	**10.1**	127	9.5	32	4.6			
Sometimes	710	73.6	911	68.1	465	67.5			
Frequently/always	158	16.4	299	22.4	192	**27.9**			
							24.906	≤.001	
Being under pressure with academic work								(p=.000)	
Not at all/slightly	136	14.1	159	11.9	88	12.8			
Fairly	499	**51.7**	674	50.4	288	41.8			
Very much	330	34.2	504	37.7	313	**45.4**			
Academic performance perception							60.038	≤.001	
Poor/reasonable	212	22.0	362	27.1	259	**37.6**			
Good	550	57.0	755	56.5	349	50.7			
Very good/excellent	203	**21.0**	220	16.5	81	11.8			
	**Difficulty falling asleep**								
	**Never/almost never (N=965)**		**Sometimes (N=1337)**		**Almost always/always(N=689)**		**F**	**p**	**Post-hoc**
	**M**	**SD**	**M**	**SD**	**M**	**SD**			
Anxiety - trait (STAI-T)	42.27	8.43	44.06	8.79	48.81	9.94	111.198	≤.001	a<b<c
Self-regulation - total	147.69	17.35	144.93	17.89	136.77	20.00	75.562	≤.001	a>b>c
Short-term self-regulation subscale	61.81	8.59	60.26	9.00	56.20	9.97	79.127	≤.001	a>b>c
Long-term self-regulation subscale	85.88	12.61	84.67	12.49	80.58	13.42	37.019	≤.001	a>c/b>c
Resilience	56.61	7.65	55.62	8.01	53.68	8.55	26.981	≤.001	a>b>c
Hours of sleep at weeknights	7.13	0.96	7.09	1.06	6.66	1.22	45.616	≤.001	a>c/b>c
Hours of sleep at weekend nights	8.44	1.25	8.60	1.24	8.57	1.63	4.376	≤.050	a<b>c
Sleep habits	3.17	1.19	4.84	1.18	5.34	1.07	855.823	≤.001	a<b<c

In bold – values that correspond to an adjusted residual ≥│1.9│.

### Factors associated with having sleeping well, a good perception of academic performance and worries in Portuguese university students

Using a binary logistic regression analysis, we obtained three adjusted models (Hosmer and Lemeshow χ²=9.325 (8), *p*=.316; χ²=82.570 (8), *p*=.186; χ²=43.370 (8), *p*=.216; respectively) and the regression equations explained 20%, 12% and 27% of the variance (Nagelkerke R2=.199; Nagelkerke R2=.124; Nagelkerke R2=.267; respectively) and 93.8% of cases that showed having sleeping well, 75.1% of cases that showed having a good perception of academic performance and 92.2% of cases that showed having worries (Table 3).

**Table 3.** Factors associated with having sleeping well, perception of good academic performance and worries in Portuguese university students (N=2,991).

	Sleeping well					Academic performance perception					Worries				
	β	SE	OR	95% IC	p	β	SE	OR	95% IC	p	β	SE	OR	95% IC	p
Hours of sleep at weeknights	.381	.075	1.464	(1.264 – 1.695)	.000	.011	.041	1.011	(0.932 – 1.097)	.789	.016	.074	1.017	(0.879 – 1.176)	.825
Hours of sleep at weekend nights	.319	.055	1.375	(1.234 –1.533)	.000	-.028	.033	.972	(0.911 – 1.037)	.394	-.086	.066	.917	(0.806 – 1.044)	.192
Sleep habits	-.575	.068	0.563	(0.492 – 0.644)	.000	-.049	.031	.952	(0.896 – 1.012)	.007	.335	.054	1.398	(1.258 – 1.554)	.000
Anxiety - trait (STAI-T)	-.042	.011	0.959	(0.939 – 0.979)	.000	-.036	.006	.964	(0.953 – 0.975)	.000	.141	.013	1.151	(1.123 – 1.180)	.000
Self-regulation - total	.004	.010	1.004	(0.986 – 1.024)	.647	.040	.005	1.041	(1.030 –1.052)	.000	-.002	.010	0.998	(0.979 – 1.017)	.806
Long-term self-regulation subscale	.003	.013	0.997	(0.972– 1.022)	.793	.038	.007	.963	(0.949 – 0.977)	.000	-.008	.014	0.992	(0.964 – 1.021)	.577
Resilience	.032	.011	0.968	(0.947 – 0.990)	.004	.018	.006	1.019	(1.007 – 1.031)	.002	-.011	.011	1.011	(0.989 – 1.034)	.320
Worries	.072	.446	1.075	(0.449 – 2.573)	.872	-.714	.165	.490	(0.354 – 0.677)	.000	-	-	-	-	-
Academic performance perception	.213	.173	0.808	(0.575 – 1.134)	.005	-	-	-	-	-	-.553	.169	0.575	(0.413– 0.802)	.001

Notes: OR: Adjusted odds ratios for all variables in the table; CI: Confidence interval.

In the first model, the condition of "having sleeping well" is explained by the variables "hours of sleep at weeknights" (1.5 times greater likelihood) [OR 1.46; 95% CI 1.26-1.70; *p*=0.000], "hours of sleep at weekend nights" (1.4 times greater likelihood) [OR 1.37; 95% CI 1.23-1.53; *p*=0.000], and sleep habits, anxiety, resilience and academic performance perception (0.6 to 1 times greater likelihood) [OR 0.56; 95% CI 0.49-0.64; *p*=0.000; OR 0.96; 95% CI 0.94-0.98; *p*=0.000; OR 0.97; 95% CI 0.95-0.99; *p*=0.004; OR 0.81; 95% CI 0.58-1.13; *p*=0.005; respectively]. This is to say that the likelihood of sleeping well is greater for those subjects who reported sleeping longer during weeknights and weekend nights, having better sleep habits, less anxiety, more resilience, and better academic performance perception.

In the second model, the condition of "having a good perception of academic performance" is explained by the variables: sleep habits, anxiety, self-regulation, long-term self-regulation, resilience, and worries. That is to say that those who reported having better sleep habits, less anxiety, more self-regulation, more long-term self-regulation, more resilience, and less worries have a 0.5 to 1 times greater likelihood of performing better academically [OR 0.95; 95% CI 0.90-1.01; *p*=0.007; OR 0.96; 95% CI 0.95-0.98; *p*=0.000; OR 1.04; 95% CI 1.03-1.05; *p*=0.000; OR 0.96; 95% CI 0.95-0.98; *p*=0.000; OR 1.02; 95% CI 1.00-1.03; *p*=0.002; OR 0.49; 95% CI 0.35-0.68; *p*=0.000; respectively].

And in the third model, the condition of "having worries" is explained by the variables: sleep habits, anxiety, and academic performance perception. Those who reported having worse sleep habits, more anxiety, and worse academic performance perception are 0.6 to 1.4 times more likely of showing worries [OR 1.38; 95% CI 1.26-1.55; *p*=0.000; OR 1.15; 95% CI 1.12-1.18; *p*=0.000; OR 0.58; 95% CI 0.41-0.80; *p*=0.000; respectively]. In other words, having worries is a negative predictor of lower academic performance perception and poor sleep habits.

### Limitations and strengths

The present results need to be interpreted keeping in mind that recall bias might be introduced through self-report, and some youths may be under-represented due to the group's heterogeneity. Furthermore, the cross-sectional design of the study precludes inferences concerning causality and longitudinal data would be needed.

However, the present study has numerous strengths, such as including self-reports from a large sample of young people, and using measures that have been shown to be suitable to assess the importance of sleep in the quality of academic life and mental health, that are validated for the Portuguese population, and that have been used in an international project -the Health Behaviour in School-aged Children (HBSC/WHO). In addition, it emphasizes the importance of sleep and how it can influence the quality of academic life, namely the academic performance perception, and mental health of college students in Portugal.

## DISCUSSION

Understanding relations between sleep, quality of academic life, namely the academic performance perception, and mental health in college students with healthy sleep habits in general is important because student sleep habits tend to worsen over time and even time-limited experience of sleep problems may have significant implications for example to the academic performance and onset of mental health problems^2,3^.

The primary goal of the present research was (a) to explore the importance of sleep habits and report the associations of sleep problems (e.g., difficulty falling asleep [insomnia]) with quality of academic life, namely academic performance perception and different dimensions of mental health (e.g., worries, anxiety, self-regulation, and resilience); (b) to determine the difficulty falling asleep (insomnia) was associated with self-reported quality of academic life and mental health in university students with general healthy sleep habits; and (c) to examine which variables are good predictors of quality of sleep ("sleeping well"), academic performance perception ("having a good perception of academic performance"), and "having worries".

The results of this study provide insight into relations between sleeping and the quality of academic life and mental health in a population of college students that is at risk of sleep problems^16-19^. As such, the findings are relevant not only to students but also to university counselors and stakeholders who have an interest in promoting student health.

One of the main objectives was to assess the prevalence of poor sleep habits among college students in Portugal. Results showed most young people report average values sleep habits (M=4.41; SD=1.46) and that they sleep an average of 7 hours a week when, according to the recommendations, the ideal would be to sleep between 7h to 9h a day^6^.

This study also found that sleep problems were frequent in college students in Portugal, because results showed that more than half of these young people report to be affected by difficulties in falling asleep (insomnia) (67.7%). These Portuguese numbers were considerably higher than numbers reported by other studies with Portuguese adolescents and by other countries with university students^3,8,11,12^.

The conducted analyses indicated that the difficulty in falling asleep (insomnia) was associated with poor/reasonable academic performance perception, higher levels of concerns and anxiety, lower levels of self-regulation and resilience, thus jeopardizing the mental health of college students. In turn, that characteristics of sleep was also associated with poor sleep habits.

If having difficulties falling asleep constitutes a problem for the health of young people; sleeping too much is not the solution, nor will it mitigate the effects of sleeping too little too often or missing nights of sleep, which happens quite often among college students.

Regression analyses revealed that greater sleep consistency, better sleep habits, better academic performance perception, low levels of anxiety, and higher levels of resilience were associated with "having sleeping well". A binary logistic regression revealed that these measures accounted for 20% of the variance in overall grade of sleeping well. In addition, the second regression analyses revealed that had better sleep habits, not having worries, low levels of anxiety and higher levels of self-regulation and resilience were associated with a positive academic performance perception. Thus, there was a substantial association between academic performance, sleep and mental health. The present results correlating overall sleep habits and mental health with academic performance perception are well aligned with previous studies^1-5,22-26^ on the role of sleep and mental health on cognitive performance.

Also, our results suggest that sleep problems may have more far reaching effects on self-regulation and resilience. In addition, the stability of sleep problems and self-regulation and resilience over time also indicates the need for early interventions to reduce sleep disorders and subsequent daytime dysfunction, thereby improving students' ability to adapt; this is uniquely important considering the high-pressure environment of Portuguese Universities.

According some studies, greater self-regulation and resilience is associated with a variety of positive adult outcomes, such as better physical health, more financial security, less criminality, and less substance use, and it is possible that improving early self-regulation and resilience may promote health and functioning over the life course^24-26^.

Our data suggest that sleep-related variables contribute to better self-regulation and resilience, and future research should prospectively evaluate the impact of multiple dimensions of sleep on the development of self-regulation and resilience. Although it is possible that poor self-regulation and poor resilience could contribute to unhealthy sleep practices that in turn result in sleep deficits. Self-regulation and resilience in college students contributes to a range of positive health and functioning outcomes that have potential long-term implications. The development of self-regulation and of resilience during youth may be adversely affected by exposure to sleep-related stressors, such as the circadian misalignment associated with evening chronotype and increased sleepiness, both of which may be related to the timing and duration of nighttime sleep. Efforts to optimize youth nighttime sleep, including implementation of healthy university start times and education on healthy sleep habits, may mitigate these effects.

In conclusion, this study showed that poor sleep habits were associated with worse levels of academic performance perception and low levels of mental health among college students in Portugal.

In light of the demonstrated associations between poor sleep and poor academic performance and mental health problems in this and other research, future studies should be conducted to examine how existing sleep interventions impact academic performance and mental health, both in students with good or poor academic performance and in students with sleep problems and with healthy sleep habits. There is a growing body of literature examining the efficacy of sleep interventions in college populations, with most interventions focused on psychoeducational sleep programs, mindfulness or relaxation training, and cognitive behavioral therapy^27-29^. Cognitive behavioral therapy in particular has been effective at improving nighttime sleep and mental health outcomes in individuals with and without insomnia^29^.

The high prevalence of sleep problems in college students and their strong associations with academic performance and mental health may warrant implementation of interventions to promote sleep awareness, sleep hygiene and practices within the university setting. Identifying and solving sleep problems at an early stage of young adults' life is important to improve their overall health, including mental health. Universities offer enormous potential as settings to promote sleep health since they can reach many young people who are future-oriented and willing to learn. There is then the need for academic researchers, teaching staff and health professionals working for college students' health to develop and test a wide array of sleep-promoting interventions (e.g., education classes, online programs, adjustment of class time), thus preventing negative secondary outcomes.
